# Stage-Specific Expression of Lens-Associated Structural Genes During Early Embryogenesis in European Seabass (*Dicentrarchus labrax*)

**DOI:** 10.3390/genes17050590

**Published:** 2026-05-21

**Authors:** Andreas Tsipourlianos, Nikolaos Veliotis, Rafael Angelakopoulos, Themistoklis Giannoulis, Katerina A. Moutou

**Affiliations:** 1 Department of Biochemistry and Biotechnology, University of Thessaly, Biopolis, 41500 Larissa, Greece; nveliotis@uth.gr (N.V.); rangelak@uth.gr (R.A.); kmoutou@uth.gr (K.A.M.); 2 Department of Animal Science, University of Thessaly, Greece Gaiopolis, 41334 Larissa, Greece; thgianno@uth.gr

**Keywords:** *Dicentrarchus labrax*, embryogenesis, lens-associated gene expression, crystallins, eye lens, eye development, visual ontogeny, aquaculture

## Abstract

**Background/Objectives:** Lens development is an essential component of visual-system development during fish embryogenesis, yet its transcriptional timing remains poorly characterized in European seabass (*Dicentrarchus labrax*). This study aimed to provide a stage-resolved transcriptomic characterization of lens-associated gene expression in *D. labrax* embryos and to relate these patterns to classical embryological stages. **Methods:** Publicly available RNA-seq data from embryos at the mid-gastrula, late somitogenesis, and hatching stages were analyzed. A targeted lens-associated gene set was defined using Gene Ontology annotations, with emphasis on genes assigned to the structural constituent of the eye lens category. Expression patterns were examined using normalized counts, variance-stabilized data, principal component analysis, and pairwise differential expression analysis. **Results:** Lens-associated genes displayed clear stage-dependent expression dynamics. Principal component analysis separated samples primarily by developmental stage, with the first two components explaining 89.3% of the total variance. The strongest biological shift occurred between mid-gastrula and late somitogenesis, when transcripts encoding β-crystallins and lens-fiber architecture components increased markedly. Among the most pronounced changes were the induction of *crybb1l3* and *cryba4*, along with increased expression of membrane and cytoskeletal genes, such as the *lim2* paralogs and *bfsp1*. By hatching, this structural-gene expression pattern was partly maintained, while specific crystallin-related loci, including *crybg1a*, showed further stage-associated increases. **Conclusions:** These findings define stage-specific patterns of lens-associated gene expression in *D. labrax* embryos and indicate that lens-associated structural gene expression is most pronounced during late somitogenesis among the stages analyzed. This work provides a useful reference for future studies of visual development in European seabass and for aquaculture-oriented investigations of early sensory ontogeny.

## 1. Introduction

European seabass (*Dicentrarchus labrax*) is a prominent marine finfish species in European aquaculture and is also widely used as a biological model for marine teleost research [1,2]. As with most marine finfish species, large-scale production relies on hatchery systems that supply juveniles to grow-out farms; despite major advances in hatchery technology, the embryonic and early larval period represents a critical bottleneck in marine aquaculture, as even small deviations in environmental conditions can strongly influence survival, growth, and the incidence of developmental abnormalities [3,4,5,6]. For *D. labrax*, this sensitivity is especially relevant because the transition from endogenous reserves to exogenous feeding occurs shortly after hatching and requires coordinated maturation of multiple organ systems that support sensory detection, swimming, and prey capture [7,8,9,10].

Among the systems that shape early larval performance, the visual system is particularly influential because many larval behaviors essential for survival, such as prey detection, feeding strikes, and spatial orientation, are strongly light-dependent [11]. In *D. labrax*, manipulations of the photic environment demonstrate that light spectrum and photoperiod can alter growth and survival trajectories during early rearing [12], while earlier work showed that strong light intensities can be lethal to newly hatched larvae under specific conditions [13]. Beyond performance metrics, light conditions can also influence retinal microstructure and ultrastructure in *D. labrax* larvae, indicating that the visual system is developmentally plastic during early life and potentially sensitive to hatchery regimes [14]. Together, these observations motivate a clearer understanding of when ocular components, particularly the lens, are being assembled during embryogenesis, because timing in ontogeny defines both functional readiness and potential windows of vulnerability.

The crystalline lens is the principal refractive element of the vertebrate eye and must achieve high transparency while packing extremely high concentrations of specialized proteins to generate optical power [15]. In fishes, this requirement is intensified by lifelong lens growth: the lens must function from larval stages onward even as new fiber cells are added, creating strong constraints on how lens structure and protein composition are built and maintained [16]. Developmentally, lens formation is governed by a conserved vertebrate developmental sequence in which inductive interactions and transcriptional networks establish lens-competent ectoderm and drive placode formation, invagination, and differentiation [17,18]. As differentiation proceeds, gene expression shifts toward modules that support transparency, cellular order, and homeostasis, features that are directly relevant to optical function [7].

These functional demands are mirrored in the major gene categories that define lens development and lens physiology and that are the focus of the present analysis. The first category comprises crystallins, including α-, β-, and γ-crystallins, which dominate the lens proteome and contribute to refractive index and long-term transparency [19]. Teleosts often show expanded γ-crystallin subfamilies and lineage-specific paralogs, making stage-resolved expression patterns particularly informative for understanding the mechanism governing the acquisition of the distinctive optical properties of the fish lens [20]. The second category includes lens membrane proteins, notably aquaporin-0/major intrinsic protein (*AQP0/MIP*) and lens intrinsic membrane protein 2 (*LIM2/MP20*), which contribute to water homeostasis, cell–cell adhesion, and the maintenance of lens architecture required for transparency [21,22]. The final category is essential for cytoskeletal specialization of lens fibers through beaded filament proteins such as *bfsp1* (filensin), which are lens-enriched structural elements implicated in maintaining lens integrity and optical quality [23,24].

For *D. labrax*, detailed morphological staging descriptions exist and provide a strong developmental framework, but corresponding stage-specific data on lens-associated gene expression are limited. The growing availability of publicly deposited omics datasets has created new opportunities to extract additional biological insight from existing transcriptomic resources. Reanalysis of such datasets using focused biological questions, updated gene annotation, or targeted gene-family frameworks can reveal patterns that were not necessarily addressed in the original studies. In this context, the publicly available embryonic RNA-seq datasets generated within the AQUAFAANG initiative provide a valuable opportunity to investigate developmental processes in *D. labrax* from a focused biological perspective. To our knowledge, no previous study has specifically examined the lens-associated transcriptional gene expression profile during *D. labrax* embryogenesis or interpreted these expression dynamics in relation to established morphological staging in this species. Importantly, *D. labrax* embryology explicitly reports the timing of early eye structures and lens appearance: crystalline lenses are not observed before the stage of approximately 11 somite pairs, and eye melanization is reported to occur concomitantly with the onset of exotrophic behavior [8]. Temperature-dependent developmental studies further support the use of discrete embryonic stages for comparative work in *D. labrax*, as developmental rates (and therefore the timing of organogenesis) shift with incubation conditions, even when the sequence of morphological milestones is conserved [25]. From an applied perspective, post-hatch timing is also consequential because first-feeding schedules and early rearing conditions can strongly influence larval performance and survival in *D. labrax* [7], and the photic environment can modulate feeding and locomotor behaviors in ways consistent with vision-mediated control of early life performance [11].

Using RNA-seq data, this study characterizes lens-associated gene expression across three embryonic stages and relates these molecular patterns to established morphological staging in *D. labrax*. These stages were selected to bracket the expected window of lens-related transcriptional change. Mid-gastrula was used as a pre-morphogenetic baseline, capturing an early embryonic state in which major body-patterning processes are underway. However, overt lens structures are not yet morphologically evident. Late somitogenesis was selected as the principal organogenic interval because ocular structures are progressing toward lens formation during this period, making it the most informative sampled stage for detecting the emergence of lens-associated structural gene expression. Hatching was included as a later developmental endpoint at the immediate pre-larval transition, allowing assessment of whether lens-related transcriptional changes persist, stabilize, or continue to increase after the organogenic window. In *D. labrax*, this framework is consistent with embryological descriptions, indicating that crystalline lenses are not observed before approximately 11 somite pairs [8].

By spanning these points, the design allows us to examine stage-associated transitions from earlier lens-related gene activity to later differentiation-associated expression modules dominated by crystallins and lens fiber structural components, and to assess whether hatching is accompanied by a distinct expression signature. More broadly, stage-resolved transcriptomic designs are increasingly used to characterize developmental transitions and to map gene expression dynamics in fish and other organisms [26,27,28].

Accordingly, we aim to provide a stage-resolved transcriptomic characterization of lens-associated gene expression in *D. labrax* embryos, linking classical embryological staging to lens gene activity. Specifically, we (1) quantify the expression trajectories of major lens gene classes, including crystallins, lens membrane proteins, and lens cytoskeletal components, across gastrulation, late somitogenesis, and hatching; (2) identify stage associated expression patterns that may be useful for describing lens-related transcriptional changes across *D. labrax* embryogenesis; and (3) interpret these patterns in the context of conserved vertebrate lens biology and *D. labrax* developmental timing to provide a reference for developmental and hatchery-focused studies where the photic environment and early performance are tightly coupled.

## 2. Materials and Methods

### 2.1. RNA-Seq Data and Quantification

Publicly available RNA-seq data from *D. labrax* embryos were retrieved from the European Nucleotide Archive (ENA) under BioProject PRJEB52160. The dataset comprised nine paired-end RNA-seq libraries representing three developmental stages: mid-gastrula (27 hpf; *n* = 3), late somitogenesis (72 hpf; *n* = 3), and hatching (92 hpf; *n* = 3). Libraries were generated on the Illumina NovaSeq 6000 platform (Illumina, San Diego, CA, USA) as paired-end 150 bp reads. Embryos were reared at 15 °C prior to hatching. Metadata for all analyzed samples are provided in Table A1. Raw reads were retrieved from ENA and quality assessed using FastQC v0.12.1 [29], with reports summarized using MultiQC v1.14 [30]. All samples showed high per-base quality based on QC reports (Table A2, Figure A2). Transcript abundance was quantified with Salmon v1.10.1 [31] (quasi-mapping mode) against the *D. labrax* reference transcriptome (dlabrax2021 assembly, GCA_905237075.1). Salmon mapping statistics were additionally inspected to complement the per-base quality assessment. Across the nine samples, mapping rates ranged from 59.4% to 69.2%, with mapped read counts ranging from 18.5 to 31.4 million (Figure A4). Salmon transcript-level estimates were then summarized to gene-level counts for downstream normalization, differential expression analysis, and targeted lens-gene interpretation. Transcript identifiers and gene annotation were obtained from the corresponding Ensembl GTF for *D. labrax*, which was also used to generate transcript-to-gene mapping files for gene-level summarization.

### 2.2. Differential Expression Analysis

Transcript quantifications from Salmon were imported into R v4.3.2 [32] and summarized to gene-level counts using tximport v1.28.0 [33]. Differential expression analysis was performed in DESeq2 v1.40.2 [34] using gene-level counts and median-of-ratios size-factor normalization. No additional user-defined low-count filtering step was applied prior to inference. Differential expression analysis was performed using the standard DESeq2 negative-binomial framework on gene-level counts. Pairwise comparisons included late somitogenesis vs. mid-gastrula, hatching vs. mid-gastrula, and hatching vs. late somitogenesis. Genes were considered differentially expressed at padj < 0.05 (Benjamini–Hochberg correction) and |log2FC| > 0.5. Differential expression testing was performed at the gene level across the full set of quantified genes. The lens-associated gene set was used subsequently for targeted interpretation, visualization, and reporting.

### 2.3. Lens-Associated Gene Set, Annotation, and Visualization

A lens-associated gene set was defined using Gene Ontology annotations. Genes annotated to “structural constituent of eye lens” (GO:0005212) were retrieved for *D. labrax* using g:Profiler [35] and used for targeted downstream interpretation. The GO:0005212 category provides a defined set of genes encoding structural constituents of the eye lens, including crystallins and lens fiber-associated components. This annotation-based selection was used to generate a focused gene set for downstream expression profiling across developmental stages. Genes are reported using stable *D. labrax* Ensembl gene identifiers, with symbols where available. For loci lacking informative symbols, *D. labrax* IDs were queried in g:Profiler to identify *Danio rerio* orthologs; the zebrafish gene name was used as an explanatory label when a confident ortholog was returned. Genes that remained unnamed after orthology mapping were additionally inspected using DAVID v2025_2 [36] to obtain descriptive functional terms where possible. Paralog designations, including those used for *lim2*-related genes and crystallin family members, followed the corresponding Ensembl gene annotations and Ensembl paralogy predictions; these labels were used to distinguish multiple annotated loci within expanded gene families rather than to infer new evolutionary relationships. In all cases, the original *D. labrax* stable ID was retained in tables and figures. The analyzed lens-associated genes, together with the displayed gene labels used in the manuscript and their annotation sources, are listed in Table A4. Stage-associated expression patterns were summarized using DESeq2-normalized counts and variance-stabilizing transformed (VST) values. Heatmaps were generated using (i) log2-transformed normalized counts [log2(count + 1)] and (ii) VST values with row-wise z-score standardization. For the selected genes, barplots show mean normalized expression per stage (μ ± SE), and significance is indicated using adjusted *p*-values from DESeq2 comparisons. Principal component analysis (PCA) was performed on both the full RNA-seq dataset and the selected lens-associated gene set using log2-transformed normalized counts. For the trajectory summary, lens-associated genes were grouped using a predefined rule-based approach based on their stage-specific mean expression profiles. For each gene, DESeq2-normalized counts from the three biological replicates within each developmental stage were averaged, and the resulting stage means were transformed as log2(mean + 1) to reduce differences in absolute expression magnitude among genes. Genes were then assigned to one of three temporal expression-pattern groups according to the relative ordering of their stage-specific mean expression values: (i) late somitogenesis peak, when late somitogenesis > mid-gastrula and late somitogenesis > hatching; (ii) progressive increase, when mid-gastrula ≤ late somitogenesis ≤ hatching; and (iii) progressive decrease, when mid-gastrula ≥ late somitogenesis ≥ hatching. Genes that did not match any of these predefined patterns were classified as (iv) mixed/other and were not included in the grouped trajectory visualization.

## 3. Results

### 3.1. RNA-Seq Dataset Quality Summary

Nine paired-end RNA-seq libraries were analyzed, with sequencing depths ranging from 30 to 45 million reads per sample. GC content was highly consistent across libraries (48–49%), and all samples passed the evaluated quality-control criteria. Duplication levels were broadly comparable among libraries, and no substantial adapter or overrepresented sequence issues were detected. Detailed quality-control metrics for individual sequencing files are provided in Table A2 and Figure A2. A whole-transcriptome PCA based on log2-transformed normalized counts showed overall separation of samples primarily by developmental stage, with no obvious within-stage outliers (Figure A3).

### 3.2. Stage-Associated Expression of Lens Structural Genes

**Figure 1:**
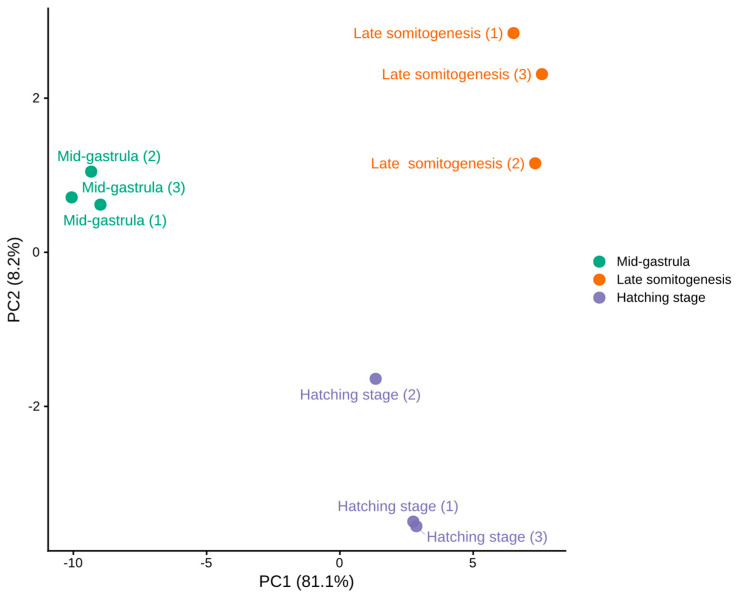
Principal component analysis of the selected lens-associated gene set expression across three embryonic stages of *D. labrax*. PCA was performed on log2-transformed normalized counts for the selected lens-associated gene set across mid-gastrula, late somitogenesis, and hatching (*n* = 3 biological replicates per stage). Each point represents one sample, and samples are colored by developmental stage.

**Figure 2:**
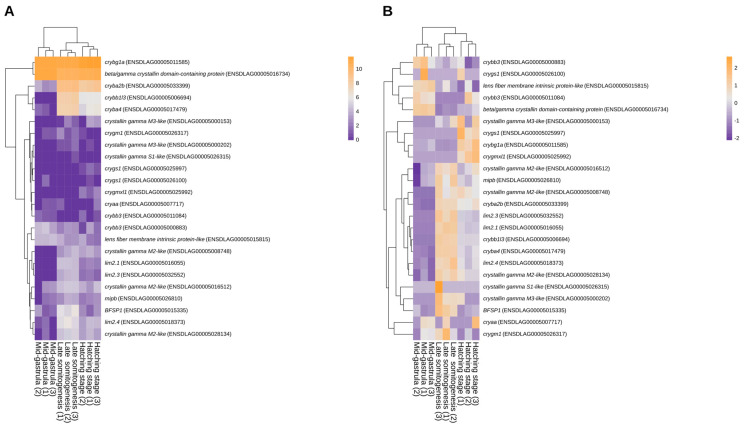
Developmental shift of the lens-associated gene expression in *D. labrax* embryos. (**A**) Heatmap of log2-transformed normalized counts (log2[count + 1]) for the lens-associated gene set. Rows correspond to genes and columns to individual embryo samples. Color intensity reflects expression magnitude. (**B**) Heatmap of variance-stabilizing transformed (VST) expression values z-scored per gene (row-wise) to emphasize relative within-gene temporal trajectories across stages.

Lens-associated genes displayed clear stage-dependent expression patterning across mid-gastrula, late somitogenesis, and hatching (Figure 1 and Figure 2, Table A3). The lens-associated gene set spans major structural components of the lens, including crystallins (α, β, and γ families), membrane proteins important for lens fiber physiology (e.g., *mipb* and *lim2* paralogs), and the lens fiber cytoskeletal element *bfsp1*. PCA of this gene set separated samples primarily by developmental stage, with mid-gastrula clustering apart from late somitogenesis and hatching; the first two principal components (PC1 and PC2) explained 89.3% of the total variance.

Across development, the expression landscape is consistent with progressive activation of lens structural genes (Figure 2A). Core β-crystallin genes, *cryba2b* (ENSDLAG00005033399), *cryba4* (ENSDLAG00005017479), and *crybb1l3* (ENSDLAG00005006694) are most prominent at late somitogenesis and/or hatching relative to mid-gastrula. A similar stage-linked increase is evident for genes associated with lens fiber architecture and homeostasis, including *mipb* (ENSDLAG00005026810), *lim2* paralogs (*lim2.1* ENSDLAG00005016055; *lim2.3* ENSDLAG00005032552; *lim2.4* ENSDLAG00005018373), and *bfsp1* (ENSDLAG00005015335), together with several *crystallin gamma M2-like* loci (ENSDLAG00005008748, ENSDLAG00005016512, ENSDLAG00005028134). Two genes show comparatively high expression across all stages: *crybg1a* (ENSDLAG00005011585) and a *beta/gamma-crystallin-domain-containing* gene (ENSDLAG00005016734). In contrast, *crybb3* (ENSDLAG00005000883) and the *lens fiber membrane intrinsic protein-like* gene (ENSDLAG00005015815) reach their highest levels at mid-gastrula, persist into late somitogenesis, and then decline moderately by hatching.

A subset of lens-associated genes showed very low abundance across all stages, consistent with strong tissue dilution and/or highly restricted spatiotemporal expression. This low-expression cluster was dominated by several γ-crystallin entries, including multiple “*crystallin gamma M3-like*” loci (ENSDLAG00005026317, ENSDLAG00005000153, ENSDLAG00005000202, ENSDLAG00005001953, ENSDLAG00005026315, ENSDLAG00005025997, ENSDLAG00005026100, ENSDLAG00005025992) and included *cryaa* (ENSDLAG00005007717) and *crybb3* (ENSDLAG00005011084).

### 3.3. Temporal Trajectories Revealed by Within-Gene Standardization

To compare temporal patterns independent of overall expression magnitude, variance-stabilized expression values were standardized within each gene (row-wise z-scores) and visualized as a heatmap (Figure 2B). Samples remained separated primarily by developmental stage, with mid-gastrula clustering apart from the later stages and late somitogenesis grouping closer to hatching. At the gene level, three distinct patterns were apparent, which were consistent with the developmental stages under study.

In detail, the mid-gastrula stage exhibited higher expression of two *crybb3* loci (ENSDLAG00005000883 and ENSDLAG00005011084), the *lens fiber membrane intrinsic protein-like* gene (ENSDLAG00005015815), and the *crystallin-domain–containing* gene (ENSDLAG00005016734). In contrast, multiple core lens structural and architectural genes, along with several γ-crystallin genes, were up-regulated during late somitogenesis. Finally, the hatching stage exhibited increased expression of *crybg1a* (ENSDLAG00005011585), *crygs1* (ENSDLAG00005025997), *crygmxl1* (ENSDLAG00005025992), and a *crystallin gamma M3-like* locus (ENSDLAG00005000153).

### 3.4. Differential Gene Expression Between Developmental Stages

**Figure A1:**
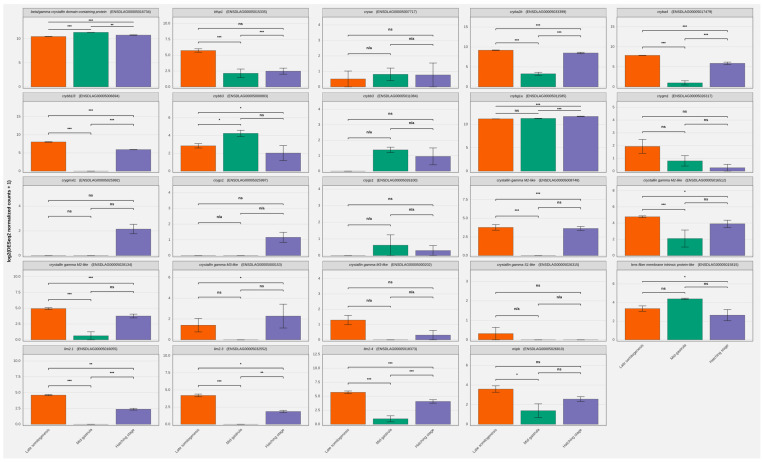
Differential expression and stage-associated abundance of selected lens structural genes during *D. labrax* embryogenesis. Barplots show mean normalized gene expression (DESeq2 size-factor–normalized counts) ± standard error (SE) across the three developmental stages (mid-gastrula, late somitogenesis, and hatching; *n* = 3 biological replicates per stage). For each gene, statistical significance of pairwise stage comparisons was assessed using DESeq2 on raw counts, and adjusted *p*-values (Benjamini–Hochberg) are indicated by asterisks above the corresponding comparisons. Asterisks denote significance thresholds as follows: *p*adj < 0.05 (*), <0.01 (**), and <0.001 (***); “ns” indicates not significant.

**Figure A2:**
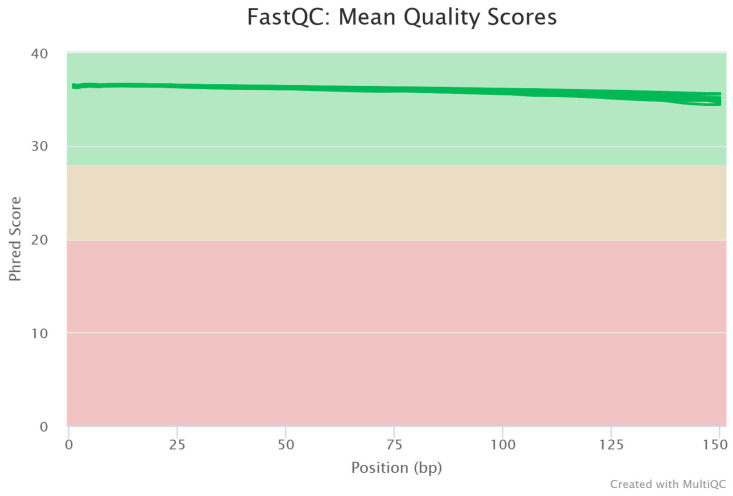
Per-base sequence quality of RNA-seq reads. MultiQC summary of FastQC mean Phred quality scores across read positions for all libraries. Quality remained consistently high across the full 150 bp read length, with all samples showing mean scores above Q30 and only a minor decrease near the read end.

**Figure A3:**
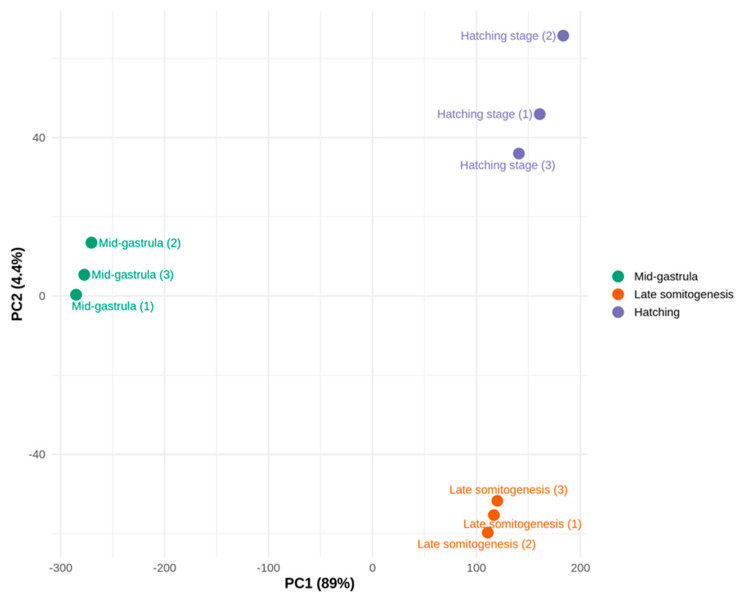
Whole-transcriptome principal component analysis of embryonic RNA-seq samples. PCA was performed on log2-transformed normalized counts for all quantified genes across the nine *D. labrax* embryo libraries. Each point represents one biological replicate, and samples are colored by developmental stage. Samples grouped primarily by developmental stage, with no obvious within-stage outliers.

**Figure A4:**
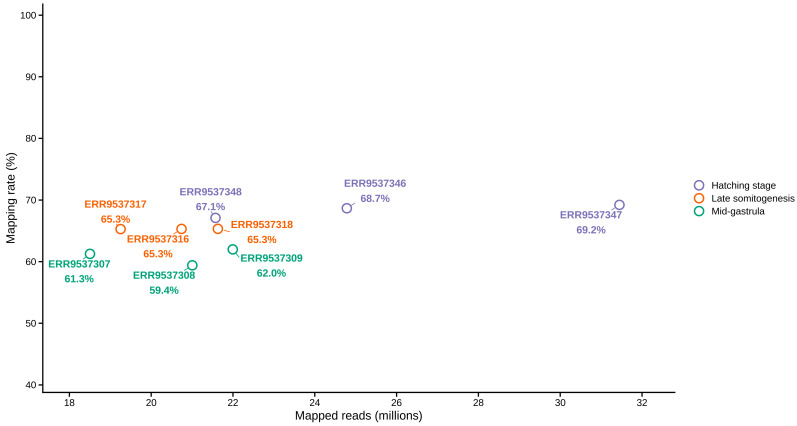
Salmon mapping summary for the analyzed embryonic RNA-seq libraries. Each point represents one *D. labrax* sample and shows the relationship between mapped read count (millions) and Salmon mapping rate (%). Samples are colored by developmental stage (mid-gastrula, late somitogenesis, and hatching). Mapping rates ranged from 59.4% to 69.2%, with mapped read counts ranging from 18.5 to 31.4 million across the nine libraries.

Differential gene expression revealed a pronounced developmental shift in lens structural gene expression across stages (Table 1; Figure A1). The direction and magnitude of each pairwise contrast are reported as DESeq2 log2 fold changes in Table 1, while adjusted *p*-values indicate statistical support after multiple-testing correction. From mid-gastrula to late somitogenesis, strong inductions were observed for β-crystallin genes *cryba2b* (ENSDLAG00005033399), *cryba4* (ENSDLAG00005017479), and *crybb1l3* (ENSDLAG00005006694), together with lens fiber structural and membrane genes *bfsp1* (ENSDLAG00005015335), *mipb* (ENSDLAG00005026810), and *lim2* paralogs (*lim2.1* ENSDLAG00005016055; *lim2.3* ENSDLAG00005032552; *lim2.4* ENSDLAG00005018373). Several γ-crystallin–annotated loci were also increased significantly by late somitogenesis, including *crystallin gamma M2-like* genes (ENSDLAG00005008748, ENSDLAG00005028134, ENSDLAG00005016512). In contrast, the *crystallin-domain–containing* gene (ENSDLAG00005016734) was significantly lower at later stages relative to mid-gastrula, and *crybb3-like* (ENSDLAG00005000883) and the *lens fiber membrane intrinsic protein-like* gene (ENSDLAG00005015815) showed overall downward trends from mid-gastrula toward later stages with significant differences in relevant comparisons. In the hatching vs. late somitogenesis contrast, multiple genes that peaked at late somitogenesis declined significantly by hatching, most notably *crybb1l3* (ENSDLAG00005006694), *cryba4* (ENSDLAG00005017479), *cryba2b* (ENSDLAG00005033399), *bfsp1* (ENSDLAG00005015335), and the *lim2* paralogs (ENSDLAG00005016055, ENSDLAG00005032552, ENSDLAG00005018373) whereas *crybg1a* (ENSDLAG00005011585) increased significantly from late somitogenesis to hatching. Somitogenesis represents the strongest elevation for many membrane/cytoskeletal and several crystallin genes, while hatching retains high expression for a subset, but with gene-specific increases or partial declines.

## 4. Discussion

### 4.1. Stage-Resolved Patterns of Lens-Associated Gene Expression

Early embryogenesis in *D. labrax* involves coordinated transitions in the expression of genes required for tissue differentiation and organ formation. Focusing on a lens-associated gene set, our analyses revealed distinct temporal patterns across mid-gastrula, late somitogenesis, and hatching, consistent with progressive establishment of lens structural and architectural features. These findings place lens gene activity within an embryological context and provide a basis for interpreting stage-specific molecular events during *D. labrax* development.

**Figure 3:**
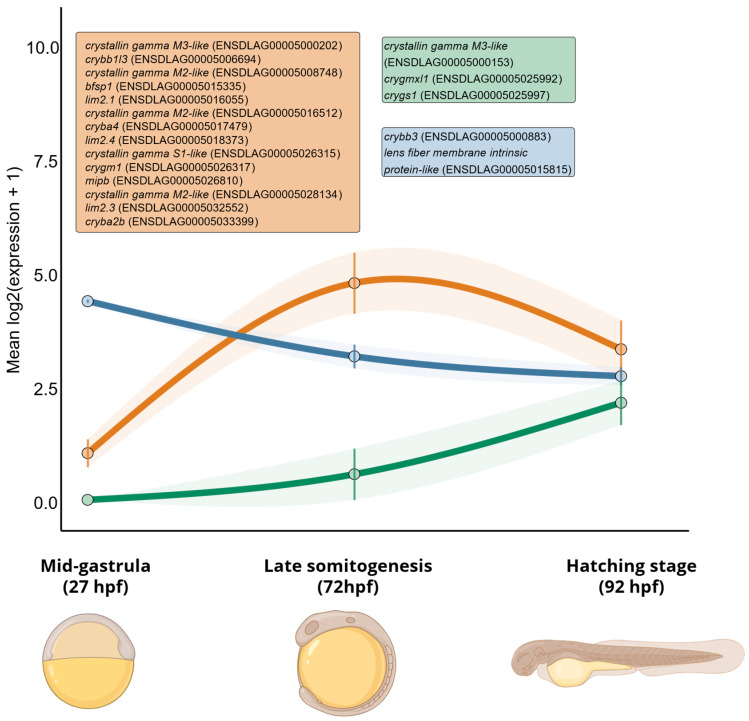
Temporal expression trajectories of lens-associated structural genes across the selected developmental stages. Expression values of the selected genes were analyzed across the three sampled developmental stages and summarized into three predefined temporal expression-pattern groups based on the relative ordering of their stage-specific mean expression values: (i) late-somitogenesis peak, when late somitogenesis > mid-gastrula and late somitogenesis > hatching; (ii) progressive increase, when mid-gastrula ≤ late somitogenesis ≤ hatching; and (iii) progressive decrease, when mid-gastrula ≥ late somitogenesis ≥ hatching. For each group, lines show the mean expression profile displayed using natural spline interpolation, and shaded areas indicate the standard error of the mean. Orange, green, and blue trajectories indicate late-somitogenesis peak, progressive increase, and progressive decrease groups, respectively. Colored labels identify the genes assigned to each group. Stage representations were adapted from BioRender to illustrate the corresponding developmental time points.

The observed stage-associated expression patterns indicate that mid-gastrula precedes the strong expression of many lens structural genes. In contrast, late somitogenesis and hatching capture the sampled interval in which these genes become more prominent. The coordinated increase in β-crystallins (*cryba2b*, *cryba4*, *crybb1l3*, and *crybb3* paralogs), together with membrane and cytoskeletal components associated with lens fiber organization (*mipb*, *lens fiber membrane intrinsic protein-like*, *lim2.1*, *lim2.3*, *lim2.4*, and *bfsp1*), supports late somitogenesis as the strongest sampled stage for lens-associated structural gene activation in *D. labrax*. At the same time, γ-crystallin-related loci showed heterogeneous expression patterns, suggesting that members of this expanded gene family are not transcriptionally deployed uniformly during the sampled embryonic window. The trajectory analysis summarized these patterns into three broad temporal profiles: (i) genes peaking at late somitogenesis, (ii) genes increasing progressively toward hatching, and (iii) genes decreasing across the sampled stages. These profiles provide a compact visualization of the main expression patterns described above and highlight the distinction between genes with a transient late-somitogenesis peak and genes with sustained or increasing expression toward hatching (Figure 3).

### 4.2. Biological Interpretation

The present study provides a stage-specific view of lens-associated gene expression in *D. labrax* embryos and is consistent with a pattern in which lens-associated structural-gene expression is most evident during the organogenesis phase, followed by gene-specific consolidation at hatching. The strongest signal in our targeted lens gene set was the coordinated upregulation from mid-gastrula to late somitogenesis of β-crystallins (*cryba2b*, *cryba4*, *crybb1l3*) together with lens fiber structural and membrane-associated components (*bfsp1*, *mipb*, and multiple *lim2* paralogs). This combination is biologically coherent because crystallin accumulation must be coupled to the establishment of a highly ordered fiber-cell architecture and specialized membrane organization to achieve transparency and high refractive power. In vertebrates, lens clarity depends on minimizing light scatter through tight control of cellular packing and protein organization, and both membrane and cytoskeletal specializations contribute to that “biological glass” state [15]. The observed timing is also consistent with *D. labrax* embryological staging, where crystalline lenses are not reported before the somitogenesis period, around the appearance of multiple somite pairs, indicating that the structural-gene signal detected here aligns with the known morphological progression in *D. labrax* [8]. The differential responses between late somitogenesis and hatching refine this interpretation and suggest a transition from an active assembly window toward a more stabilized developmental state as embryos approach the pre-larval phase. Several genes that rose strongly by late somitogenesis then declined significantly by hatching, including *bfsp1* and the *lim2* paralogs, whereas *crybg1a* showed a significant increase into the hatching stage. This pattern fits a modular lens-development logic: a temporally concentrated phase in which membrane and cytoskeletal components are maximally expressed to build and organize the fiber mass, followed by continued crystallin tuning as the optical protein environment matures. *bfsp1* is a lens-specific intermediate filament component with established roles in lens fiber structure and optical maintenance, and changes in its expression are consistent with shifting requirements for cytoskeletal remodeling during differentiation [24]. Similarly, *mipb* contributes not only to water homeostasis but also to cell–cell adhesion properties important for lens organization and transparency, making its developmental induction a biologically expected hallmark of lens fiber maturation [21]. Together, these comparisons suggest that late somitogenesis represents an important stage for lens-associated structural gene expression in *D. labrax*, while hatching captures a subsequent phase characterized by selective persistence or further increases in specific crystallin components.

### 4.3. Comparative Context and Implications

Previous studies with teleost model systems are in agreement with the observed patterns in *D. labrax* in terms of lens biology and highlight the complexity of crystallin deployment within expanded fish crystallin repertoires. Teleost γ-crystallins comprise multiple related loci, and such gene-family expansion can provide the substrate for divergence in expression timing, abundance, and potentially lens-region or developmental-stage specificity. Single-cell atlases in zebrafish demonstrate that lens gene expression is highly dynamic and that crystallin family members can be deployed with distinct temporal and cell-type specificity across early development [37]. Comparable trends are also described in Xenopus, where crystallin transcripts increase during the onset of lens fiber differentiation and subsequently stabilize as development progresses [38], and in mouse, where embryonic to perinatal lens maturation is accompanied by extensive remodeling of crystallin, membrane, and cytoskeletal gene expression [39]. In the present study, some γ-crystallin-related loci, particularly *crystallin gamma M2-like* genes, increased significantly by late somitogenesis and/or hatching. In contrast, several other γ-crystallin-related loci remained at very low abundance across the sampled stages. This heterogeneous pattern is consistent with differential regulation among members of an expanded γ-crystallin family and may indicate functional or developmental partitioning among loci. However, because the present analysis used whole-embryo RNA-seq and annotation-based gene labels, these data should be interpreted as evidence for stage-associated transcriptional divergence among γ-crystallin-related loci, rather than as direct proof of functional divergence. Beyond basic developmental insight, these results are important for aquaculture because visual system maturation is tightly linked to early larval performance and to environmental sensitivity, particularly to the photic environment. In *D. labrax*, light spectrum and photoperiod influence growth and survival across early life, and larval retinal structure shows measurable responses to light conditions, emphasizing that visual-system development is plastic and potentially vulnerable to husbandry regimes [12,14]. The stage-resolved trajectories identified here provide useful transcriptional reference patterns for future experiments testing whether hatchery-relevant factors shift the timing or magnitude of lens-associated gene activation, especially around late somitogenesis, where lens-associated architectural gene expression was most evident within the sampled developmental window. Although whole-embryo sampling may limit the detection of low-abundance or spatially restricted transcripts, the consistency of the stage-associated patterns across biological replicates supports the main transcriptional trends observed during the sampled embryonic window.

### 4.4. Limitations

Several limitations of the present study should be considered when interpreting the results. First, the analysis was based on whole-embryo RNA-seq data rather than lens-isolated or tissue-resolved transcriptomic data. Because the lens represents only a small fraction of the embryo, tissue dilution is likely to reduce the detectability of low-abundance or spatially restricted transcripts and to limit the ability to attribute observed expression changes specifically to lens tissue rather than to broader embryonic processes. Second, the study did not include independent validation by qPCR or spatial approaches such as in situ hybridization, and the reported expression patterns should therefore be regarded as transcriptomic associations that provide a focused reference for future confirmation. Third, only three developmental stages were analyzed; these provide discrete temporal snapshots but do not resolve finer transitions within the interval between mid-gastrula and late somitogenesis. Accordingly, the present results are most informative for identifying stage-associated changes across the sampled embryonic window. In contrast, denser temporal sampling and tissue-resolved approaches will be required to refine the timing and tissue specificity of lens-related transcriptional events in *D. labrax*.

## 5. Conclusions

This study provides a targeted, stage-resolved molecular framework for lens-associated gene expression in *D. labrax* that complements established morphological staging and identifies a coherent activation sequence across three key embryonic milestones. By integrating normalized expression patterns, differential expression contrasts, and gene-family-specific trajectories, it provides a practical reference for European seabass development: late somitogenesis emerges as the strongest sampled stage for the expression of multiple lens structural and architectural modules. Meanwhile, hatching reflects consolidation with gene-specific continuation of crystallin accumulation. As with most whole-embryo developmental RNA-seq designs, interpretation is limited by tissue heterogeneity and by the discrete sampling of three stages; future work using denser staging around somitogenesis and tissue-resolved approaches will help refine lens-specific timing and separate lens-driven expression from broader embryonic processes. Nonetheless, by extracting lens-focused information from a publicly available embryonic RNA-seq resource, the present study fills an important gap in the stage-resolved molecular characterization of early visual system development in *D. labrax*. It provides a useful reference for future tissue-resolved validation, comparative developmental studies, and aquaculture-oriented investigations.

## Figures and Tables

**Table 1:** Differential expression of lens-associated genes across embryonic stages in *D. labrax*. Values represent DESeq2 log2 fold changes for pairwise stage comparisons. Asterisks denote statistically significant contrasts based on adjusted *p*-values (Benjamini–Hochberg correction; * *p*adj < 0.05, ** *p*adj < 0.01, *** *p*adj < 0.001). Dashes indicate non-significant contrasts. Differential expression was defined using a |log2FC| > 0.5.

Gene ID	Gene Symbol/Annotation	Late vs. Mid	Hatch vs. Late	Hatch vs. Mid
ENSDLAG00005033399	*cryba2b*	+5.92 ***	−0.69 ***	+5.24 ***
ENSDLAG00005017479	*cryba4*	+7.54 ***	−1.97 ***	+5.57 ***
ENSDLAG00005006694	*crybb1l3*	+10.13 ***	−2.13 ***	+7.99 ***
ENSDLAG00005000883	*crybb3*	−1.58 *	—	−2.10 *
ENSDLAG00005011585	*crybg1a*	—	+0.52 ***	—
ENSDLAG00005028134	*crystallin gamma M2-like*	+5.15 ***	—	+3.97 ***
ENSDLAG00005008748	*crystallin gamma M2-like*	+5.87 ***	—	+5.67 ***
ENSDLAG00005016512	*crystallin gamma M2-like*	+2.36 ***	—	+1.57 *
ENSDLAG00005000153	*crystallin gamma M3-like*	—	—	+4.77 *
ENSDLAG00005016734	*beta/gamma crystallin domain-containing protein*	−0.86 ***	—	−0.56 ***
ENSDLAG00005015815	*lens fiber membrane intrinsic protein-like*	—	—	−1.57 *
ENSDLAG00005032552	*lim2.3*	+6.25 ***	−2.72 **	+3.53 *
ENSDLAG00005018373	*lim2.4*	+5.44 ***	−1.71 ***	+3.73 ***
ENSDLAG00005015335	*bfsp1*	+3.52 ***	−3.40 ***	—
ENSDLAG00005026810	*mipb*	+2.45 *	—	—

## Data Availability

The RNA-seq datasets analyzed in the present study are publicly available in the European Nucleotide Archive (ENA) under BioProject accession PRJEB52160. The analyzed *D. labrax* embryo samples correspond to the developmental stages mid-gastrula (27 hpf), late somitogenesis (72 hpf), and hatching (92 hpf), with run accessions ERR9537307–ERR9537309, ERR9537316–ERR9537318, and ERR9537346–ERR9537348, respectively.

## Appendix A

**Table A1:** Metadata for the *D. labrax* embryonic RNA-seq samples used in this study. Sample identifiers, European Nucleotide Archive (ENA) run accessions, BioSample IDs, developmental stages, and sampling times (hours post-fertilization, hpf) are shown for the nine libraries analyzed across mid-gastrula, late somitogenesis, and hatching.

Sample ID	ENA Run Accession	BioSample ID	Developmental Stage	Time (hpf)
mid-gastrula_1	ERR9537307	SAMEA12116057	mid-gastrula	27
mid-gastrula_2	ERR9537308	SAMEA12116064	mid-gastrula	27
mid-gastrula_3	ERR9537309	SAMEA12116072	mid-gastrula	27
late_somitogenesis_1	ERR9537316	SAMEA12116184	late somitogenesis	72
late_somitogenesis_2	ERR9537317	SAMEA12116193	late somitogenesis	72
late_somitogenesis_3	ERR9537318	SAMEA12116201	late somitogenesis	72
hatching_1	ERR9537346	SAMEA12116263	hatching	92
hatching_2	ERR9537347	SAMEA12116271	hatching	92
hatching_3	ERR9537348	SAMEA12116278	hatching	92

**Table A2:** Summary of per-sample RNA-seq quality metrics for the *D. labrax* paired-end libraries.

Sample Name	% Dups	% GC	M Seqs
ERR9537307_1	63.40%	48%	30.2
ERR9537307_2	62.00%	48%	30.2
ERR9537308_1	63.40%	48%	35.4
ERR9537308_2	62.30%	48%	35.4
ERR9537309_1	65.30%	48%	35.5
ERR9537309_2	64.50%	48%	35.5
ERR9537316_1	62.30%	49%	31.8
ERR9537316_2	61.00%	49%	31.8
ERR9537317_1	61.20%	49%	29.5
ERR9537317_2	59.70%	49%	29.5
ERR9537318_1	63.40%	49%	33.1
ERR9537318_2	62.20%	49%	33.1
ERR9537346_1	68.60%	48%	36.1
ERR9537346_2	66.30%	49%	36.1
ERR9537347_1	68.60%	48%	45.4
ERR9537347_2	68.40%	49%	45.4
ERR9537348_1	64.30%	48%	32.1
ERR9537348_2	65.00%	48%	32.1

**Table A3:** Normalized expression values for lens-associated genes across embryonic stages in *D. labrax.*

Gene Symbol/Annotation (Gene ID)	Mid-Gastrula (1)	Mid-Gastrula (2)	Mid-Gastrula (3)	Late Somitogenesis (1)	Late Somitogenesis (2)	Late Somitogenesis (3)	Hatching Stage (1)	Hatching Stage (2)	Hatching Stage (3)
*crystallin gamma M3-like* (ENSDLAG00005000153)	0.00	0.00	0.00	0.26	4.93	1.47	11.49	0.00	8.17
*crystallin gamma M3-like* (ENSDLAG00005000202)	0.00	0.00	0.00	0.93	1.02	2.74	0.90	0.00	0.00
*crybb3* (ENSDLAG00005000883)	26.04	20.34	10.82	4.17	8.16	6.89	11.07	0.61	2.50
*crystallin gamma M3-like* (ENSDLAG00005001953)	0.00	0.00	0.00	0.00	0.00	0.00	0.00	0.00	0.00
*crygm5* (ENSDLAG00005001958)	0.00	0.00	0.00	0.00	0.00	0.00	0.00	0.00	0.00
*crybb1l3* (ENSDLAG00005006694)	0.00	0.00	0.00	255.79	241.95	286.71	59.96	56.44	63.05
*cryaa* (ENSDLAG00005007717)	1.41	0.00	1.23	1.90	0.00	0.00	0.00	0.00	3.96
*crystallin gamma M2-like* (ENSDLAG00005008748)	0.00	0.00	0.00	7.61	13.46	20.06	16.23	11.25	8.27
*crybb3* (ENSDLAG00005011084)	1.39	2.29	1.22	0.00	0.00	0.00	0.00	2.70	0.97
*crygm5* (ENSDLAG00005011417)	0.00	0.00	0.00	0.00	0.00	0.00	0.00	0.00	0.00
*crybg1a* (ENSDLAG00005011585)	2439.71	2426.74	2592.03	2305.46	2354.12	2319.81	3302.44	3138.67	3580.50
*bfsp1* (ENSDLAG00005015335)	1.33	9.97	2.36	52.90	37.48	71.87	1.94	5.61	7.89
*lens fiber membrane intrinsic protein-like* (ENSDLAG00005015815)	19.62	18.54	22.23	14.29	7.23	7.44	3.37	13.34	2.91
*lim2.1* (ENSDLAG00005016055)	0.00	0.00	0.00	25.31	24.73	20.39	5.26	3.56	3.93
*crystallin gamma M2-like* (ENSDLAG00005016512)	7.20	0.00	8.66	22.65	29.72	27.53	7.85	14.10	24.45
*beta/gamma crystallin domain-containing protein* (ENSDLAG00005016734)	2645.67	2597.80	2537.42	1467.28	1443.51	1365.87	1669.77	1690.29	1906.72
*cryba4* (ENSDLAG00005017479)	1.40	2.31	0.00	232.89	226.65	242.27	68.09	37.98	75.68
*lim2.4* (ENSDLAG00005018373)	2.63	1.09	0.00	45.62	70.78	41.93	20.49	10.06	19.20
*crygs3* (ENSDLAG00005025990)	0.00	0.00	0.00	0.00	0.00	0.00	0.00	0.00	0.00
*crygmxl1* (ENSDLAG00005025992)	0.00	0.00	0.00	0.00	0.00	0.00	1.65	4.05	5.49
*crygs1* (ENSDLAG00005025997)	0.00	0.00	0.00	0.00	0.00	0.00	2.47	0.65	0.95
*crygs1* (ENSDLAG00005026100)	2.66	0.00	0.00	0.00	0.00	0.00	0.87	0.00	0.00
*crystallin gamma S1-like* (ENSDLAG00005026315)	0.00	0.00	0.00	0.00	0.00	0.94	0.00	0.00	0.00
*crygm1* (ENSDLAG00005026317)	1.41	0.00	1.21	6.49	1.02	2.73	0.00	0.74	0.00
*mipb* (ENSDLAG00005026810)	2.79	0.00	3.68	6.62	14.37	13.86	5.08	3.37	6.81
*crystallin gamma M2-like* (ENSDLAG00005027880)	0.00	0.00	0.00	0.00	0.00	0.00	0.00	0.00	0.00
*crystallin gamma M2-like* (ENSDLAG00005028134)	2.74	0.00	0.00	24.21	32.47	33.45	19.62	10.19	9.89
*lim2.3* (ENSDLAG00005032552)	0.00	0.00	0.00	13.08	19.29	21.01	3.40	2.71	1.94
*cryba2b* (ENSDLAG00005033399)	5.61	15.19	7.48	570.76	556.36	632.66	345.26	296.80	454.08
*crystallin gamma M3-like* (ENSDLAG00005034722)	0.00	0.00	0.00	0.00	0.00	0.00	0.00	0.00	0.00

**Table A4:** Lens-associated genes analyzed in *D. labrax*, including Ensembl gene identifiers, displayed gene labels and annotation sources.

Ensembl Gene ID	Display Name	Annotation Source
ENSDLAG00005000153	*crystallin gamma M3-like* (ENSDLAG00005000153)	DAVID
ENSDLAG00005000202	*crystallin gamma M3-like* (ENSDLAG00005000202)	DAVID
ENSDLAG00005000883	*crybb3* (ENSDLAG00005000883)	zebrafish ortholog
ENSDLAG00005001953	*crystallin gamma M3-like* (ENSDLAG00005001953)	DAVID
ENSDLAG00005001958	*crygm5* (ENSDLAG00005001958)	zebrafish ortholog
ENSDLAG00005006694	*crybb1l3* (ENSDLAG00005006694)	Ensembl annotation
ENSDLAG00005007717	*cryaa* (ENSDLAG00005007717)	Ensembl annotation
ENSDLAG00005008748	*crystallin gamma M2-like* (ENSDLAG00005008748)	DAVID
ENSDLAG00005011084	*crybb3* (ENSDLAG00005011084)	zebrafish ortholog
ENSDLAG00005011417	*crygm5* (ENSDLAG00005011417)	zebrafish ortholog
ENSDLAG00005011585	*crybg1a* (ENSDLAG00005011585)	zebrafish ortholog
ENSDLAG00005015335	*bfsp1* (ENSDLAG00005015335)	Ensembl annotation
ENSDLAG00005015815	*lens fiber membrane intrinsic protein-like* (ENSDLAG00005015815)	DAVID
ENSDLAG00005016055	*lim2.1* (ENSDLAG00005016055)	Ensembl annotation
ENSDLAG00005016512	*crystallin gamma M2-like* (ENSDLAG00005016512)	DAVID
ENSDLAG00005016734	*beta/gamma crystallin domain-containing protein* (ENSDLAG00005016734)	DAVID
ENSDLAG00005017479	*cryba4* (ENSDLAG00005017479)	Ensembl annotation
ENSDLAG00005018373	*lim2.4* (ENSDLAG00005018373)	Ensembl annotation
ENSDLAG00005025990	*crygs3* (ENSDLAG00005025990)	zebrafish ortholog
ENSDLAG00005025992	*crygmxl1* (ENSDLAG00005025992)	zebrafish ortholog
ENSDLAG00005025997	*crygs1* (ENSDLAG00005025997)	zebrafish ortholog
ENSDLAG00005026100	*crygs1* (ENSDLAG00005026100)	zebrafish ortholog
ENSDLAG00005026315	*crystallin gamma S1-like* (ENSDLAG00005026315)	DAVID
ENSDLAG00005026317	*crygm1* (ENSDLAG00005026317)	Ensembl annotation
ENSDLAG00005026810	*mipb* (ENSDLAG00005026810)	Ensembl annotation
ENSDLAG00005027880	*crystallin gamma M2-like* (ENSDLAG00005027880)	DAVID
ENSDLAG00005028134	*crystallin gamma M2-like* (ENSDLAG00005028134)	DAVID
ENSDLAG00005032552	*lim2.3* (ENSDLAG00005032552)	Ensembl annotation
ENSDLAG00005033399	*cryba2b* (ENSDLAG00005033399)	Ensembl annotation
ENSDLAG00005034722	*crystallin gamma M3-like* (ENSDLAG00005034722)	DAVID
